# Survey of major trauma centre preparedness for mass casualty incidents in Australia, Canada, England and New Zealand

**DOI:** 10.1016/j.eclinm.2020.100322

**Published:** 2020-04-02

**Authors:** Belinda J. Gabbe, William Veitch, Kate Curtis, Kate Martin, David Gomez, Ian Civil, Chris Moran, Warwick J. Teague, Andrew J.A. Holland, Fiona Lecky, Mark Fitzgerald, Avery Nathens, Anthony Joseph

**Affiliations:** a Department of Epidemiology and Preventive Medicine, Monash University, 553 St Kilda Rd, Melbourne 3004, Australia; b School of Medicine, University of Sydney, Edward Ford Building (A27) Fisher Road, University of Sydney, Sydney 2006, Australia; c Susan Wakil School of Nursing and Midwifery, University of Sydney, 88 Mallett St, Camperdown 2050, Australia; d Trauma Service, The Alfred, 55 Commercial Rd, Melbourne 3004, Australia; e Division of General Surgery, St. Michael's Hospital, Unity Health Toronto, University of Toronto, 30 Bond St, Toronto M5B 1W8, ON, Canada; f Faculty of Medical and Health Sciences, University of Auckland, 85 Park Rd, Grafton 1023, Auckland, New Zealand; g Department of Trauma and Orthopaedics, Nottingham University Hospital, Hucknall Rd, Nottingham NG5 1 PB, United Kingdom; h Trauma Service, The Royal Children's Hospital, 50 Flemington Rd, Parkville 3052, Australia; i Surgical Research, Murdoch Children's Research Institute, Flemington Rd, Parkville 3052, Australia; j Department of Paediatrics, University of Melbourne, 50 Flemington Rd, Parkville 3052, Australia; k The Children's Hospital at Westmead Clinical School, The University of Sydney School of Medicine, Faculty of Medicine and Health, Westmead 2145, Australia; l Centre for Urgent and Emergency Care Research, University of Sheffield, Western Bank S10 2TN, Sheffield, , United Kingdom; m Department of Surgery, Central Clinical School, Monash University, 99 Commercial Rd, Melbourne 3004, Australia; n Department of Surgery, Sunnybrook Health Sciences Centre, University of Toronto, 2075 Bayview Ave, Toronto M4N 3M5, ON, Canada; o Royal North Shore Hospital Clinical School, School of Medicine, University of Sydney, Kolling Building Level 7, Royal North Shore Hospital, Reserve Road, St Leonards 2065, Australia

**Keywords:** Trauma centre, Disaster preparedness, Mass casualty incident, Survey, Major trauma

## Abstract

**Background:**

Mass casualty incidents (MCIs) are increasing. Trauma centres play a key role in MCIs due to their readiness and expansive multidisciplinary expertise for injury management. Previous studies have shown deficiencies in trauma centre disaster preparedness. The aim of this study was to describe the current disaster preparedness of Major Trauma Centres (MTCs) in Australia, Canada, England and New Zealand.

**Methods:**

A cross-sectional survey of all (*n* = 82) MTCs was undertaken. The anonymous survey collected data about disaster preparedness in nine key areas. Respondents were encouraged to consult appropriately at their centre to provide an accurate representation of their centre's preparedness.

**Findings:**

Responses were received from 69 (84%) centres; 61 completed all questions. 91% had a disaster preparedness committee and 80% had an all-hazards emergency plan. 79% had held an MCI drill in the past 2 years. 54% reported a system in place to calculate maximum capacity, but testing of surge capacity was uncommon. 55% reported the presence of stored resources for an MCI and 58% had a database of staff trained in Emergency Management. 74% had a training and education plan available for staff involved in an MCI and a plan for professional debriefing of staff post-MCI, while 62% had a post-disaster employee assistance programme. Most centres had appropriate back-up communication, safety and security plans.

**Interpretation:**

The disaster preparedness of MTCs was high for communication, safety and security but there was clear need for improvement in other areas including surge capacity, human resources and post-disaster recovery.

**Research in context**

***Evidence before this study***

**Trauma centres provide a critical service in mass casualty incidents, with appropriate responses requiring established and effective disaster preparedness planning. Previous studies of the disaster preparedness of trauma centres has been limited to single centres and single countries, and no recent data are available.**

***Added value of this study***

**This is the first multi-national survey of the disaster preparedness of major trauma centres. All major trauma centres in four high-income countries were invited to participate and a high response rate was achieved. The survey was designed using existing tools of the World Health Organization and previous studies. Data were captured across nine key areas.**

***Implications of all the available evidence***

**The study revealed the need for greater engagement of trauma leadership roles in institutional disaster planning, a focus on real-world disaster drills, enhanced assessment and testing of surge capacity of key specialties, accurate and timely information about staff capacity and training for disasters, and improved uptake of post-disaster plans. Improved preparedness of trauma centres will be needed to optimise responses to the growing number of mass casualty incidents occurring worldwide.**

Alt-text: Unlabelled box

## 1 Introduction

Mass casualty incidents (MCIs) result from multiple causes, including transport, mass gatherings, armed conflicts, terrorism, biological, geophysical and hydro-meteorological disasters. These events are characterised by a “quantity, severity, and diversity of injuries and other patients that can rapidly overwhelm the ability of local medical resources to deliver comprehensive and definitive medical care” [1]. Globally, the incidence and nature of MCIs varies [2], and there is an expectation that hydro-meteorological disasters will increase due to extreme weather events associated with climate change [3]. In 2018 alone there were 315 natural disasters, affecting 68.5 million people, causing 11,804 deaths, and costing the world economy $132 billion (USD) [4]. From 2000, there was a surge in terrorist attacks resulting in MCIs [5], and the recent Christchurch Mosque shootings in New Zealand (NZ) and Colombo bombings in Sri Lanka show these incidents continue to devastate communities and cause significant mortality and long-term morbidity.

The timing and nature of MCIs are unpredictable, yet they require a rapid and often sustained response by healthcare systems. Trauma centres are central to regionalised trauma care and provide a critical resource in regional responses to MCIs due to their constant state of readiness and expansive multidisciplinary expertise in injury management. Established protocols and procedures at trauma centres for MCIs are a key component of emergency preparedness, resilience and response.

Despite the expectation of readiness for MCIs, studies have found that trauma centre preparedness for disasters is sub-optimal [6], [7], [8]. Corrigan et al., in their study of disaster knowledge, preparedness and willingness to respond in a single Australian trauma centre found that while 59% of participants had received disaster preparedness education, 38% had attended a simulation drill and 13% had responded to a real-life disaster [6]. Most felt “not really” prepared or “unsure” about their preparedness to respond to a disaster [6]. Gomez et al. completed a cross-sectional survey of Canadian Level 1 trauma centres in 2009 and found that 43% had not conducted a recent disaster drill, 52% had an all hazards emergency plan, 59% were unsure if they could sustain peak operation for 72 h or more, and 61% had plans for increasing surgical capacity [7]. Lewis et al. surveyed 80 surgeons’ knowledge of the their trauma centre's MCI plan in the US and found that while 86% knew their hospital had an MCI plan, only 41% offered training in MCI preparedness, 64% had a mechanism for moving patients out of the ED and ICU in an MCI, and 50% knew whether their trauma centre had been involved in a previous MCI [8]. Traub et al. surveyed the surge capacity of 88 hospitals with emergency departments (ED) [9]. They found that Australasian hospitals would be quickly overwhelmed by multiple casualties, 60–80% of seriously injured patients would not have immediate access to operating theatres in a moderate to severe MCI, and these access issues would extend to intensive care units (ICU) and radiology facilities for less critically injured patients [9].

More recently, Moran and Brohi shared the findings of debriefings from multiple MCIs in the United Kingdom in 2017 [10]. They highlighted the importance of desktop and simulation exercises for testing plans and informing policy, as well as the need to prepare for the prolonged impact of these events on resourcing and staff [10]. Nevertheless, knowledge about the current capacity of trauma centres to respond to MCIs is lacking. Therefore, the aim of this study was to assess the preparedness for MCIs of Major Trauma Centres (MTCs, Level 1 trauma centre or equivalent) in Australia, Canada, England and NZ.

## 2 Methods

### 2.1 Study design and setting

An online, anonymous, cross-sectional survey of adult and paediatric MTCs in Australia, England, NZ and Canada was undertaken. The four countries were chosen due to similarity in the organisation and funding of healthcare with universal healthcare systems, comparable health care spending per capita, and similarity in the organisation of trauma systems and the profile of major trauma seen. The study was conducted with the endorsement of the Australasian Trauma Society (ATS) and the Trauma Association of Canada (TAC).

### 2.2 Participants

All designated or accredited MTCs (Level 1 trauma centres) in Australia (*n* = 24), NZ (*n* = 7), England (*n* = 27) and Canada (*n* = 24) were invited to participate. Trauma Directors and Trauma Coordinators (where present) at all eligible centres were invited to participate. Completion of the survey was considered consent, and ethics approval was granted by the Monash University Human Research Ethics Committee.

### 2.3 Survey and procedures

The survey was developed using the World Health Organization (WHO) Europe toolkit and checklist for assessing health-system capacity for crisis management (WHO 2008) [11,12]. This toolkit is based on an “all-hazards approach” and reflects both the planning and physical abilities of a trauma centre to be ready to respond to a major disaster. The study by Gomez et al. (2011) of Canadian Level 1 trauma centres [7], and the Surge Capacity for People in Emergencies (SCOPE) study [9], were also considered in survey development. Questions across nine key areas were included:

(i) Leadership and governance
(ii) Communication
(iii) Safety and security
(iv) Triage
(v) Surge capacity
(vi) Continuity of essential services
(vii) Human resources
(viii) Logistics and supply management
(ix) Post-disaster recovery.

The electronic survey (Supplementary Material) was distributed using Qualtrics Insight Platform software (Qualtrics, Provo, Utah, USA) in December 2018 and the survey was closed in April 2019. The survey took approximately 30 min to complete online, although this estimate does not include time spent gathering information in preparation for completion. Participants were invited to take part by email, which explained the study and provided the link to the survey. A single survey link was provided for each centre to prevent multiple responses from the same institution. Participants from Australia, Canada and NZ also received a letter of support for the study from the President of their relevant trauma society and those in England received a similar letter from their National Clinical Director for Trauma. The respondents were able to complete the survey in stages, allowing partial completion and saving, to reduce respondent burden and maximise the potential for accurate completion. Respondents were encouraged to consult with their colleagues in completing the survey to provide an accurate representation of their institution's disaster preparedness. Four reminders to complete the survey were sent; one a week after the initial invitation, and then at 2- to 3-week intervals.

### 2.4 Data analysis

Data were downloaded from the Qualtrics site for analysis in Stata Version 15 (StataCorp, College Station, Texas, USA). Frequencies and percentages were used for categorical variables, and missing responses were excluded from the calculations.

### 2.5 Role of the funding source

No funding was received for the conduct of this study.

## 3 Results

Due to consistency in responses between countries, combined data are presented here. Where differences between countries were observed, these are noted in the relevant section.

### 3.1 Respondents

Eighty-four percent (69/82) of invited centres participated; 21 (88%) Australian, 15 (63%) Canadian, 26 (96%) English, and 7 (100%) NZ centres. All survey questions were completed by 88% (61/69) of centres. The Trauma Director was the most common respondent (38/69, 55%), followed by the Trauma Coordinator (18/69, 26%), another person (9/69, 13%), Trauma Director and Trauma Coordinator (2/69, 3%) and the Trauma Director with another person (2/69, 3%).

### 3.2 Leadership and governance

#### 3.2.1 Disaster preparedness committees and all-hazards emergency plans

A committee dedicated to disaster preparedness was present at most centres (61/67, 91%) (Table 1). Most NZ centres (4/7) reported the absence of a committee. Where a committee was present, the Trauma Director was a member at 61% (37/61) of centres; 31% (19/61) expressed concern regarding adequate representation on the committee. Eighty-one percent (53/66) of centres had an all-hazards emergency plan (Table 1); ranging from 60 to 100% across countries. Where an all-hazards plan was present, 58% (31/53) had activated the plan; 17 in the previous two years.

**Table 1:** Leadership and governance, communication, triage, and safety and security characteristics for disaster preparedness planning.

Leadership and governance	Yes	No	Don't Know
Is there a committee dedicated to disaster preparedness in your institution?	61 (91%)	6 (9%)	0 (0%)
Does your institution have a single all-hazards emergency plan?	53 (80%)	7 (11%)	6 (9%)
Has your institution had a practice drill for a mass casualty event in the last 2 years?	52 (79%)	14 (21%)	0 (0%)
**Communication**	**Yes**	**No**	**Don't know**
Do you have reliable and sustainable primary backup communication systems?	54 (84%)	8 (13%)	2 (3%)
Is there access to an updated contact list?	55 (87%)	3 (5%)	5 (8%)
Is there a procedure for appointing a public information spokesperson to coordinate trauma centre communication with public, media and health authorities?	59 (94%)	1 (1%)	3 (5%)
Is there a procedure for briefing hospital staff on their roles and responsibilities with the emergency management plan?	59 (92%)	3 (5%)	2 (3%)
**Triage**	**Yes**	**No**	**Don't know**
Does your institution have a mass casualty triage protocol that follows internationally accepted principles and guidelines?	61 (97%)	1 (2%)	1 (2%)
Does your institution have a contingency site for receipt and triage of mass casualties?	48 (76%)	12 (19%)	3 (5%)
Does your institution have mechanisms in place for identifying victims and tracking missing persons?	47 (75%)	10 (16%)	6 (9%)
**Safety and security**	**Yes**	**No**	**Don't know**
Does your institution's plan include appointment of a hospital security team?	58 (93%)	1 (2%)	3 (5%)
Does your institution's plan include procedures for reliable identification of authorised hospital personnel, patients and visitors?	55 (87%)	8 (13%)	0 (0%)
Does your institution's plan include procedures for early control of facility access points, triage sites and other areas of patient flow, traffic and parking?	57 (89%)	5 (8%)	2 (3%)
Can you limit visitor access as appropriate?	57 (89%)	4 (6%)	3 (5%)
Does your institution have an established area to deal with radioactive, biological and chemical decontamination and isolation?	56 (89%)	6 (9%)	1 (2%)

#### 3.2.2 Practice drills for mass casualty incidents

**Fig. 1:**
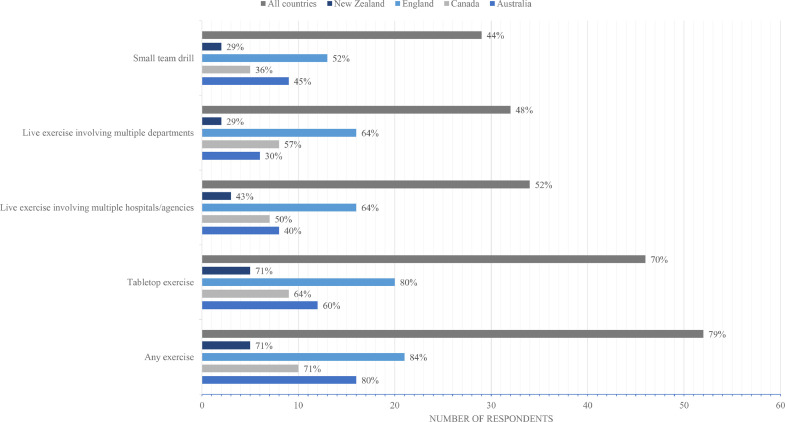
Types of disaster exercises and drills undertaken at Level 1 trauma centres in the previous two years - overall and by participating country (percentages as a calculation of total respondents and respondents by country).

Seventy-nine percent (52/66) of centres had held a mass casualty drill in the past 2 years (Table 1). Tabletop exercises were most prevalent, and 48% (32/66) had conducted multi-agency/multi-hospital drills in the past 2 years (Fig. 1). Emergency medical services (*n* = 37), police (*n* = 25), other trauma centres (*n* = 22), and the fire department (*n* = 18) were the most common external agencies engaged for live drills. Engagement of Red Cross (*n* = 5), search and rescue (*n* = 3), military (*n* = 3), and coast guard (*n* = 1) was uncommon. Mutual aid agreements or memorandums of understanding with other healthcare organisations, military, government organisations, or non-governmental organisations regarding disaster planning and cooperation during an MCI were present at 58% (37/64) centres, although the existence of agreements was unknown at 20% (13/64) of centres. Military agencies were invited to participate in training of staff at only 13/64 centres; England (10/25) and Australia (3/19). Most (50/52) centres who had undertaken practice drills reported on the impact; 80% (*n* = 40) reported that findings from the planning exercises had been incorporated into an updated disaster preparedness plan, 4% (*n* = 2) responded no, and 16% (*n* = 8) were unsure of the influence on their disaster plan.

### 3.3 Communication

**Fig. 2:**
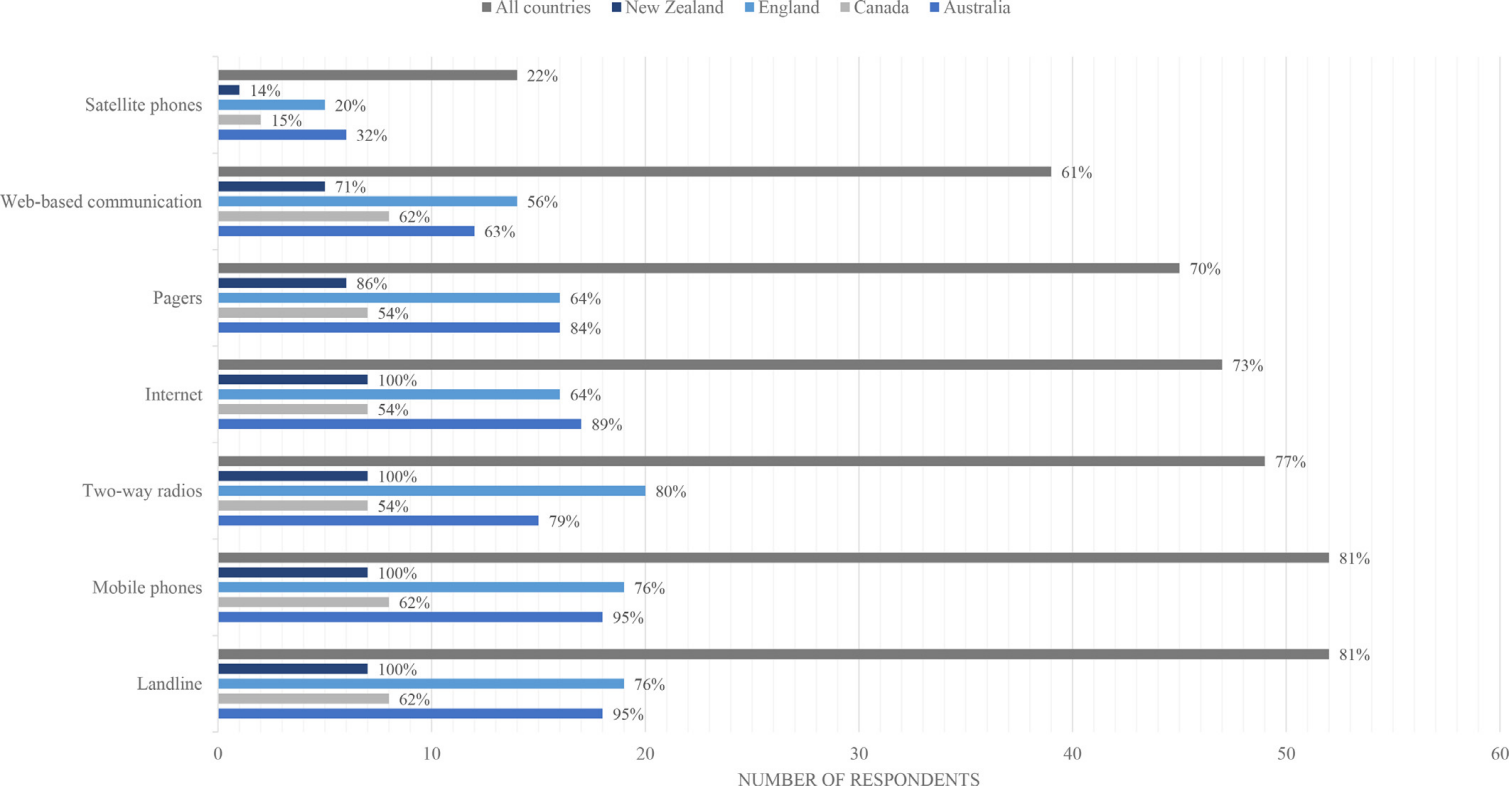
Reliable back-up communication types at Level 1 trauma centres – overall and by participating country (percentages as a calculation of total respondents and respondents by country).

Most centres (84%, 54/64) had reliable and sustainable backup communications (Table 1). Landlines and mobile phones were the most common (81%, 52/64) followed by two-way radios (77%, 49/64), internet (73%, 47/64), pagers (70%, 45/64) and web-based communication (61%, 39/64). Only 22% (14/64) of centres had a satellite phone (Fig. 2). Most reported access to an updated contact list, a procedure for appointing a public information spokesperson, and a plan for briefing hospital staff on their roles and responsibilities (Table 1).

### 3.4 Triage

Two centres (located in Australia and Canada) did not have a mass casualty triage protocol that follows internationally accepted principles and guidelines (Table 1). Seventy-six (48/63) percent had a contingency site for receipt and triage of mass casualties, and 75% (47/63) had mechanisms for victim identification and tracking.

### 3.5 Safety and security

Most (93%, 58/63) centres’ plans included a hospital security team. Procedures for reliable identification of authorised hospital personnel, patients and visitors were common (87%, 55/63), while 89% (57/64) reported the presence of procedures for early control of access points, triage sites, traffic and parking. The capacity to limit visitor access and lock-down the facility was reported by 89% (57/64) of centres, and only 10% (6/63) of centres did not have an established area to deal with radioactive, biological and chemical decontamination (Table 1).

### 3.6 Surge capacity

**Fig. 3:**
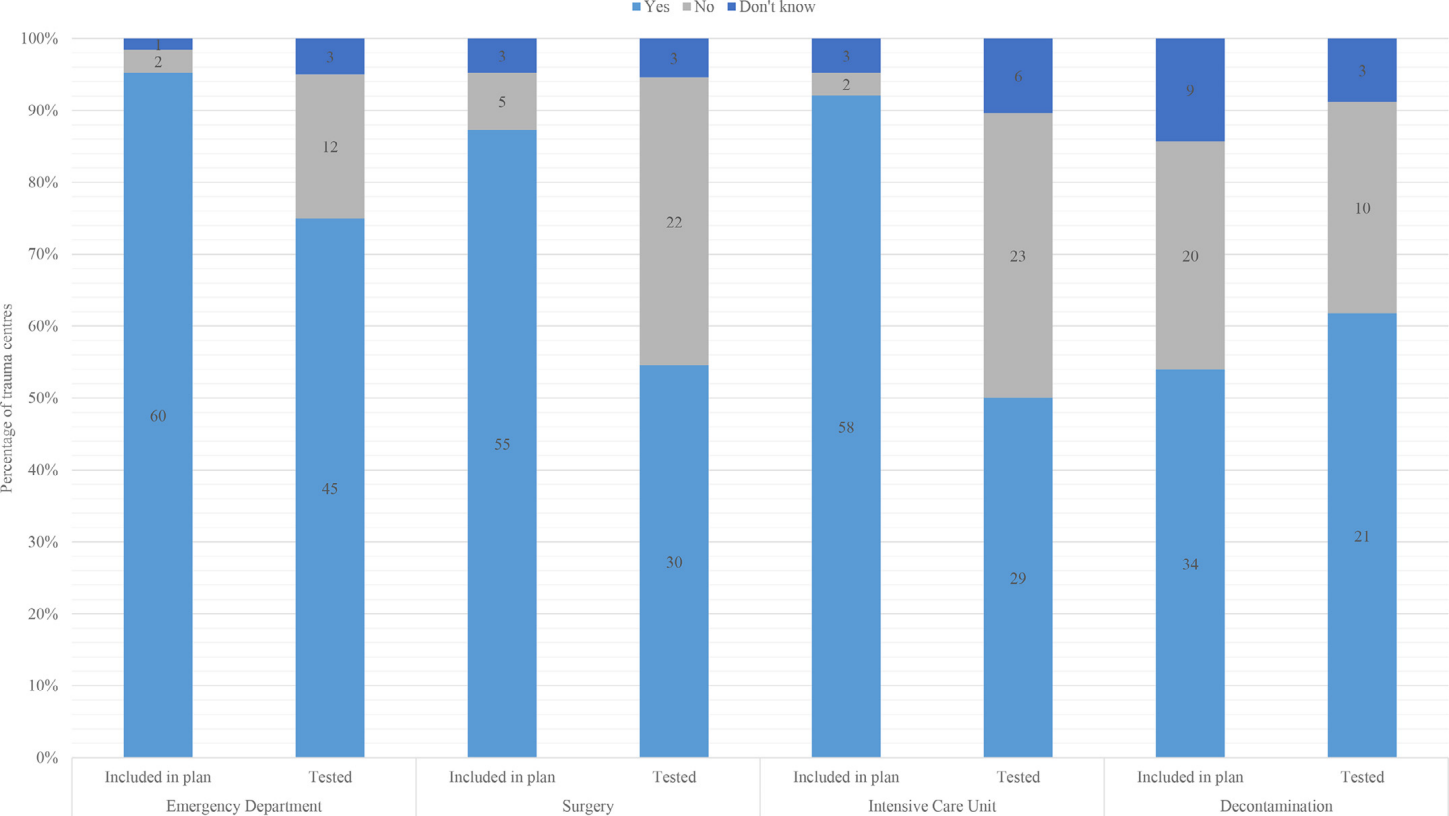
Prevalence of Major Trauma Centres who have included, and tested, surge capacity by key specialties (bar labels are the number of centres).

Most centres’ plans addressed the need for increased capacity in ICU, ED and surgery. Decontamination capacity was less commonly included, and least prevalent in Canadian centres (2/13). Where centres had included surge capacity in their plan, ED capacity was most commonly tested (Fig. 3).

Fifty-four percent (34/63) of centres had a system to calculate maximum capacity which included total bed numbers, human and essential resource needs, and adaptability of space for critical care, and this was more common in England (68%, 17/25), compared to Australia (39%, 7/18), Canada (54%, 7/13) and NZ (43%, 3/7). Of the 34 centres with surge capacity estimates, the calculation predominantly included ICU (*n* = 33), ED (*n* = 31), total beds (*n* = 30), and operating theatres (*n* = 19). Inclusion of staff availability (*n* = 19), patient tracking (*n* = 16), air medical services (*n* = 10), and early warning systems were less common.

Most (89%, 57/65) centres’ plans had designated care areas for patient overflow, including outsourcing the care of non-critical patients to other treatment sites (Table 2). Seventy-five percent (47/63) of centres reported the capacity to sustain operations at maximum occupancy for 72 h or more during an MCI (Table 2).

**Table 2:** Surge capacity, continuity of essential services, human resources, and logistics and supply management in disaster preparedness planning.

Surge capacity	Yes	No	Don't know
Does your institution's plan have designated care areas for patient overflow?	57 (89%)	5 (8%)	2 (3%)
Does your institution have a system to increase hospital capacity by outsourcing the care of non-critical patients to appropriate alternative sites?	54 (86%)	7 (11%)	2 (3%)
Does your institution have a contingency plan for interfacility patient transfer should traditional methods of transportation become available?	42 (67%)	17 (27%)	4 (6%)
Can your institution sustain operations at maximum occupancy for 72 h or more during a mass casualty event?	47 (75%)	3 (5%)	13 (20%)
**Continuity of essential services**	**Yes**	**No**	**Don't know**
Does your institution have availability of appropriate back-up arrangements for essential life lines including water, power and oxygen?	50 (81%)	4 (6%)	8 (13%)
Does your institution have stored resources in case of a mass casualty event?	34 (55%)	20 (32%)	8 (13%)
Does your institution have an established mechanism for accepting donation of materials during a mass casualty event?	7 (11%)	38 (61%)	17 (27%)
Does your institution have a system in place for determining and storing the optimal amount of pharmaceuticals, laboratory, operating equipment and blood products for a mass casualty event?	32 (52%)	18 (29%)	12 (19%)
**Human resources**	**Yes**	**No**	**Don't know**
Does your institution have a database of staff trained in Emergency Management?	36 (58%)	17 (27%)	9 (15%)
Does your institution have a training and education plan available for staff involved in mass casualty situations?	45 (74%)	12 (20%)	4 (6%)
Does your institution have a system for recruiting and training additional staff according to anticipated need?	28 (46%)	25 (41%)	8 (13%)
Does your institution have a system to ensure the availability of multidisciplinary psychosocial support teams that include social workers, counsellors, interpreters and clergy for the families, staff and patients?	52 (85%)	7 (12%)	2 (3%)
**Logistics and supply management**	**Yes**	**No**	**Don't know**
Has your institution developed and maintained an updated inventory of all equipment, supplies and pharmaceuticals?	39 (64%)	10 (16%)	12 (20%)
Does your institution have a system to ensure the continuous provision of essential medications and supplies during a mass casualty event?	45 (75%)	5 (8%)	10 (17%)
Does your institution have contingency agreements with vendors to ensure the procurement and prompt delivery of equipment, supplies and other resources in times of shortage?	40 (66%)	7 (11%)	14 (23%)

### 3.7 Continuity of essential services

Eighty-one percent (50/62) had the availability of appropriate back-up arrangements for essential resources (Table 2); 18 reported back-up arrangements lasting three or more days (Table 2). Eleven percent (7/62) of centres had an established mechanism for accepting donated materials during an MCI (Table 2).

### 3.8 Human resources

Fifty-eight percent (36/62) had a database of staff trained in Emergency Management (Table 2); only 2/13 Canadian centres responded in the affirmative. Where a database was present, this was maintained in 83% (30/36) of cases. Physicians/surgeons (32/32, 100%) and nursing staff (32/32, 100%) were most commonly included on the database, followed by hospital management (27/32, 84%), administrative staff (25/32, 78%), bed managers (23/32, 72%), allied health (20/32, 63%), security (19/32, 59%), ancillary staff (13/32, 41%), and engineering (11/32, 34%).

**Table 3:** Key recommendations for improving major trauma Centre preparedness for mass casualty incidents.

***Leadership and governance***
All-hazards emergency plans should be implemented at all major trauma centres.
Trauma medical directors should be actively engaged in disaster preparedness plans at an institutional and regional level.
Disaster preparedness planning should be a mandatory component of hospital accreditation and adequately supported by relevant governments and agencies to enable reliable, meaningful and sustainable preparation.
Multi-agency and multi-hospital drills should be regularly scheduled and regionally standardised.
***Communication***
Trauma centres should review their communication strategies to ensure sustainable and reliable communication strategies
Multi-interface interoperable communication systems should be made available at major trauma centres.
Regular review of communication strategies should be undertaken to ensure best practice in the context of rapid technology changes.
***Safety and security***
Hospital security is essential during a mass casualty response and the current high level of engagement of security teams in planning for these events must continue.
Regular review of hospital security and their procedures for lock-down and reliable identification of authorised hospital personnel, patients and visitors must be undertaken.
***Triage***
Annual review of the mass casualty triage protocol is necessary.
***Surge capacity***
Annual update of surge capacity to incorporate changes in infrastructure and transport service delivery models is needed.
***Continuity of Essential Services***
Intelligent stockpiling of resources capable of lasting at least 72 h should occur.
***Human Resources***
A complete and updated database of staff trained in Emergency Management should be maintained to facilitate optimal staffing in mass casualty incidents.
The disaster preparedness plan should extend to requiring development of department-specific plans with a graded level of response and staffing, and a priori designation of roles and responsibilities.
***Logistics and supply management***
Major trauma centres should have an updated inventory of all equipment, supplies and pharmaceuticals
Contingency agreements with vendors and suppliers must be in place to ensure continuity of supplies
Trauma centres should establish a mechanism for accepting donation of materials during a mass casualty event
***Post-disaster recovery***
Disaster preparedness plans should include plans for de-briefing and post-action reports.
Employee assistance programs should be available in the post-disaster period.
Disaster preparedness plans should address the need for prolonged treatment requirements and mental health needs of patients.

Eight-five percent (52/61) of centres reported a system for providing multidisciplinary psychosocial support teams for families, staff and patients during an MCI, 74% (45/61) had a training and education plan available for staff involved in an MCI, while less than half (48%, 28/61) reported the presence of a system for recruiting and training additional staff according to anticipated need (Table 2).

### 3.9 Logistics and supply management

Sixty-four percent (39/61) had an inventory system for equipment, supplies and pharmaceuticals, 75% (45/60) had a system to ensure the continuous provision of essential medications and supplies during an MCI, and 66% (40/61) had contingency agreements with vendors to ensure timely provision of resources in times of shortage. “Don't know” responses were common for these questions (Table 2).

### 3.10 Post-disaster recovery

Eighty-one percent (48/59) of centres’ plans included a post-action report to hospital administration, emergency managers and appropriate stakeholders that includes an incident summary, a response assessment, and an expenses report. Seventy-five percent (45/61) had a plan for professional debriefing of staff within 24–72 h of an MCI. All (7/7, 100%) NZ centres, and most (15/18, 83%) Australian centres, had a post-disaster employee recovery assistance programme compared to 8/22 (36%) English centres, and 7/13 (54%) Canadian centres.

## 4 Discussion

Trauma centres must play a critical role in MCIs and disasters due to the co-location of key clinical specialties supported by the necessary staff, processes and equipment to provide high quality care to the injured. Disaster planning and preparedness of trauma centres are fundamental to the regional response to MCIs. In this multi-national study, we investigated the disaster preparedness of MTCs in four high-income countries. The findings demonstrate widespread commitment to preparedness in all countries surveyed, but also identified need for improvement in many areas of disaster preparedness. Key recommendations are summarised and discussed here.

Strong leadership and governance ensures the presence of relevant policies and guidelines, allocation of necessary resources and accountability. These attributes are important for disaster preparedness [12]. A disaster preparedness committee coordinates emergency-preparedness planning and responses, and this was present at most participating centres. Nevertheless, the Trauma Director was not always a member, and many respondents expressed concern regarding adequate representation on the committee. Given the role of trauma centres and the frequent need for a scalable response to care for a large influx of injured patients, the absence of the Trauma Director on the planning committee of more than a third of participating centres represents a lost opportunity to integrate a trauma response into the larger organisational response to an MCI.

Disaster drills and exercises generate evidence about what does and does not work in disaster response. Periodic disaster drills are recommended. When carried out well, they allow assessment of performance in response, and refinement of procedures [12], [13], [14]. There are multiple methods for disaster drills. Real-world practice exercises are favoured over tabletop exercises [13], as tabletop exercises do not stress a system's resources sufficiently to test response. In our study, most centres had conducted a mass casualty drill in the past two years, but less than half had conducted a multi-agency/multi-hospital drill. A previous Canadian study found less than half of surveyed centres had conducted a drill [7], while only 38% of survey respondents at an Australian trauma centre had participated in a drill, either real-time or tabletop [6]. Lewis et al. reported that 86% of respondents reported that their trauma centre practised its MCI plan [8], but the type of drill or simulation was not explored. Real-world practice exercises have previously identified logistics and knowledge gaps that were undetected by discussion-based or tabletop exercises, and there is evidence of improved knowledge, perceptions and attitudes to disaster preparedness through real-world participation [14]. A key benefit of drills and exercises is the opportunity to inform and improve disaster preparedness plans, and, importantly, most participants in our study reported direct impact of planning exercise findings on their institution's plan.

Accurate and timely communication is essential in any MCI response [12]. While most participating centres reported reliable and sustainable back-up communications, none reported multi-interface interoperable communication systems and only one in five centres had a satellite phone. Most centres reported reliance on landlines, mobile phones, and pagers. The nature of MCIs can make these standard communication methods unavailable or ineffective due to physical infrastructure failure, overloading of systems or system shut-down for security reasons [15], [16], [17]. As variability in nature of the MCI will impact on different modes of communication, trauma centres require access to overlapping modes of communication. Communication within the trauma centre, as well as between the trauma centres and external agencies (e.g. emergency services and government), and with the community, is critical in responding to an MCI. Dependency on standard communication methods could place trauma centres at risk of communication failure, further compounding the challenges of delivering high quality care in a disaster. Ensuring access to multiple modes of communication which rely on different types of infrastructure, and are compatible with key external agencies, would minimise the risk of communication failure in an MCI, and should be factored into the trauma centre disaster preparedness planning.

Surge capacity represents a hospital's ability to expand beyond normal capacity to meet increased demand and provide care to critical and non-critical mass casualties simultaneously [9,12]. Adequate surge capacity implies that each centre has the ability to meet the treatment needs of the victims of the incident including resuscitation in the ED, timely and appropriate access to imaging and operating theatres, and availability of ICU beds. The resources needed will vary depending on the type of MCI. Nevertheless, an infinite capability cannot be expected and all centres have a limit beyond which the number and/or resource requirements of mass casualties overwhelms even planned for surge capacity. Most participating centres had addressed the need for surge capacity in the ICU, ED and surgery in their disaster preparedness plans, but surge capacity for decontamination in the case of a chemical, biological, radiation or nuclear event was uncommon. Additionally, testing of surge capacity was less common and most often only involved the ED, which represents a key risk to the overall response of centres to an MCI. One in five centres were unsure if they could maintain maximum capacity for 72 h, reflecting an overall level of uncertainty about surge capacity. This uncertainty persists despite identification of substantial deficiencies in surge capacity in Australian hospitals more than a decade ago [9]. As surge capacity is a marker of the ability to deliver high quality acute care in a disaster situation, the findings from this study show significant room for improvement.

Trauma centres normally operate close to capacity and need to continue to care for these patients until arrangements for transfer/discharge can be made. This requires uninterrupted supplies of essential services, equipment, supplies and pharmaceuticals. Previous natural disasters have highlighted the vulnerability of supply lines for essential resources, long delays in restocking, and the potential for functional collapse [18,19]. Most participating centres confirmed the presence of back-up arrangements, although less than half of the centres’ back-up arrangements could last 72 h or more. Careful planning and a risk-based approach is recommended to ensure appropriate purchasing decisions and the capacity to meet the anticipated needs. Consideration of the storage costs, regular inventory taking, and replacement of outdated or damaged resources is needed in the plan [20].

The availability of appropriately trained personnel, and effective human resource management to ensure continuity of services is critical in any trauma centre response to an MCI [12]. Prior studies have shown low levels of staff education and training in disaster preparedness; 26% of ED consultants in Italy had attended at least one course [21], and 59% of staff at a single Australian trauma centre reported receiving training in disaster preparedness [6]. Similarly, a survey of surgeons in the US reported that 41% of their trauma centres offered training in MCI preparedness by someone with “hands-on” experience in an MCI or disaster simulation [8]. The WHO toolkit recommends that training and education programmes in Emergency Management are available, accessible, appropriate and effective, cater to relevant clinical disciplines. These should be academically supported, accredited and curricula reviewed on a regular basis [12]. Most participating centres (74%) had training and education systems in place, but only 58% kept a database of staff trained in Emergency Management, and less than half had a system for recruiting additional staff in times of need. Military engagement in trauma centre staff training was low despite the expertise inherent in military organisations in responding to MCIs and disasters. Without sufficient records of staff trained in Emergency Management, and systems for recruiting additional staff when needed, trauma centres are at risk of insufficient trained staff to respond, staff responding without up-to-date training, or staff responding without the fundamental skills required for providing care in an MCI.

A key component of disaster response is ensuring the disaster's medium- and long-term impact on hospital operations are minimised [12]. A post-action report, staff de-briefing and establishing employee assistance programmes are important elements of the post-disaster recovery phase. Most participating centres’ disaster plans included provision of a post-action report, and 75% had a plan for debriefing staff. There were marked differences between countries with regards to access to employee assistance programmes as part of post-disaster preparedness planning. While employee assistance programmes were virtually universal in NZ and Australian centres, less than half of Canadian and English centres had such programmes in place. Moran and Brohi summarised the combined findings of debriefing processes following multiple MCIs in the United Kingdom and highlighted the need to plan for prolonged treatment requirements and mental health needs of patients, as well as support of staff, as key learnings from their experiences [10].

There were clear strengths to this study. Multiple countries were simultaneously surveyed. While the included countries have not experienced the high incidence of MCIs observed by other countries, the lower frequency of MCIs increases the importance of maintaining readiness and the understanding the challenges in doing so. Where MCIs occur infrequently, there is a clear imperative to ensure that disaster preparedness and readiness to respond are maintained. As the findings pertain to the participating countries, which were all high income countries, extrapolating the findings to other jurisdictions is unlikely to be appropriate. Notwithstanding, the methods used could be extended to other jurisdictions to improve understanding of disaster preparedness of trauma centres worldwide.

All MTCs were invited and there was a high response rate. We encouraged widespread consultation with colleagues to ensure that responses reflected each Trauma Centre's preparedness, and most sites reported consultation in completing the survey. Nevertheless, there were limitations. The survey was comprised of questions used in previous studies [7,9,13], and the WHO toolkit [12], and was piloted by a small group of trauma centre coordinators and directors for acceptability and feasibility. Notwithstanding, this was a cross-sectional survey, reflecting the state of disaster preparedness at the time of survey, and therefore changes may have occurred between closure of the survey and the time of publication. Individual variation in interpretation of questions is possible, and verification of individual responses was not able to be conducted. As the survey was open to respondents for four months, it is possible that centres may have made changes between the receipt of the survey and responding. Not all respondents consulted with colleagues and some aspects of disaster preparedness may be under-estimated. While multiple reminders were sent, 16% of trauma centres did not respond. As the survey was anonymous, we cannot ascertain how these centres differed to the responders and therefore there is the potential for responder bias. Finally, an MCI requires a whole of trauma system response which could not be fully assessed in this survey of MTCs.

Trauma centres provide a critical service in MCIs, with appropriate responses requiring established and effective disaster preparedness planning. The findings of this multi-national survey of the disaster preparedness of MTCs in four countries revealed the need for greater engagement of trauma leadership roles in institutional disaster planning, a focus on real-world disaster drills, enhanced assessment and testing of surge capacity of key specialties, accurate and timely information about staff capacity and training for disasters, and improved uptake of post-disaster plans (see Table 3).

## Declaration of Competing Interest

The authors have no interests to declare.

## Appendix Supplementary materials

Image, application 1
