# Supplementary material for: Survey of major trauma centre preparedness for mass casualty incidents in Australia, Canada, England and New Zealand
Source: eClinicalMedicine. 2020 Apr 2;21:100322. doi: 10.1016/j.eclinm.2020.100322 (PMC7201027; doi:10.1016/j.eclinm.2020.100322)
Supplement: Supplementary file 1 [file mmc1.pdf]

Start of Block: Saving reminder

Q2.1 **This survey is constructed to automatically save your responses** when the current browser window is closed. The saved responses can then be re-accessed using the URL link provided in your initial introductory email.

Please utilise this feature and ask colleagues to clarify information regarding the areas of your hospital's disaster preparedness plan that you are less familiar with thereby reducing the number of "Don't know" responses.

Each trauma centre has been assigned a single link, therefore identical links may have been provided to more than one staff member. **Please avoid completing the survey on two computers at the same time** as responses may be lost or altered.

Once the survey has been submitted it can no longer be re-accessed, you will be provided with a warning page before submitting outlining this aspect of the survey.

End of Block: Saving reminder

Start of Block: Respondent Details

Q3.1 Which country is your trauma centre located?

- ○ Australia (1)
- ○ New Zealand (2)
- ○ Canada (3)
- ○ England (5)

Q3.2 Has your institution been designated as a trauma centre by a country, state, regional or provincial authority?

- ○ Yes (1)
- ○ No (2)
- ○ Don't know ***Please consult with your colleagues to avoid using this option, if possible.*** (56)

Display This Question:

If Which country is your trauma centre located? != England

Q3.3 Has your trauma centre been accredited by the Trauma Association of Canada or the Australasian College of Surgeons?

○ Yes Most recent year of accreditation (1) ____

○ No (2)

Q3.4 What is your role at the trauma centre?

▢ Trauma Director (4)

▢ Trauma Coordinator (5)

▢ Other (specify) (6) ____

End of Block: Respondent Details

Start of Block: Leadership and Governance

Q4.1 Is there a committee dedicated to disaster preparedness in your institution?

○ Yes (1)

○ No (2)

○ Don't know ***Please consult with your colleagues to avoid using this option, if possible.*** (3)

Display This Question:

If Is there a committee dedicated to disaster preparedness in your institution? = Yes

Q4.2 Is the Trauma Director a member of the disaster preparedness committee?

○ Yes (1)

○ No (2)

○ Don't know ***Please consult with your colleagues to avoid using this option, if possible.*** (3)

Display This Question:

If Is there a committee dedicated to disaster preparedness in your institution? = Yes

Q4.3 Do you believe that there is adequate and relevant stakeholder representation on the committee?

- Yes (1)
- No (2)
- Don't know ***Please consult with your colleagues to avoid using this option, if possible.*** (4)

Q4.4 Does your institution have a single all-hazards emergency management plan?

- Yes (1)
- No (2)
- Don't know ***Please consult with your colleagues to avoid using this option, if possible.*** (3)

Display This Question:

If Does your institution have a single all-hazards emergency management plan? = Yes

Q4.5 Has the all-hazards emergency management plan been activated?

- Yes (1)
- No (2)
- Don't know ***Please consult with your colleagues to avoid using this option, if possible.*** (3)

Display This Question:

If Has the all-hazards emergency management plan been activated? = Yes

Q4.6 What was the last year that the plan was activated?

____

Q4.7 Has your institution had a practice drill for a mass casualty event in the last 2 years?

- Yes (1)
- No (2)
- Don't know ***Please consult with your colleagues to avoid using this option, if possible.*** (3)

Display This Question:

If Has your institution had a practice drill for a mass casualty event in the last 2 years? = Yes

Q4.8 Check all practice drill strategies that apply:

- Tabletop exercise (1)
- Small team drill (2)
- Live exercise involving multiple departments (3)
- Multi hospital/agency (4)

Display This Question:

If Has your institution had a practice drill for a mass casualty event in the last 2 years? = Yes

Q4.9 Check all practice drill participants that apply:

- ▢ Physicians/surgeons (1)
- ▢ Allied health personnel (2)
- ▢ Nurses (3)
- ▢ Administrative staff (4)
- ▢ Hospital management (5)
- ▢ Engineers (6)
- ▢ Security (7)
- ▢ Ancillary staff (8)
- ▢ Bed Managers (9)

Display This Question:

If Has your institution had a practice drill for a mass casualty event in the last 2 years? = Yes

Q4.10 If your institution has been involved in a live mass casualty exercise with other hospitals in your community, first responders and other stakeholders, please select which of the following participated (check all that apply):

- [ ] Police department (1)
- [ ] Fire department (2)
- [ ] Coast guard (3)
- [ ] Red Cross (4)
- [ ] Urban Search and Rescue (5)
- [ ] Military base (6)
- [ ] Emergency Medical Services (Paramedics) (7)
- [ ] Other trauma centres (8)

Display This Question:

If Has your institution had a practice drill for a mass casualty event in the last 2 years? = Yes

Q4.11 Have the findings of the planning exercise/s been incorporated into an updated disaster preparedness plan?

- ○ Yes (1)
- ○ No (2)
- ○ Don't know ***Please consult with your colleagues to avoid using this option, if possible.*** (3)

Q4.12 Has your institution established mutual aid agreements (MAA) or memorandums of understanding (MOU) with other healthcare organizations, military or governmental agencies, or non-governmental organizations (NGO) regarding disaster planning and cooperation during a mass casualty event?

- ○ Yes (1)
- ○ No (2)
- ○ Don't know ***Please consult with your colleagues to avoid using this option, if possible.*** (4)

Q4.13 Has your institution made arrangements to have military agencies participate in training of staff members?

- Yes (1)
- No (2)
- Don't know ***Please consult with your colleagues to avoid using this option, if possible.*** (3)

End of Block: Leadership and Governance

Start of Block: Communication

Q5.1 Do you have availability of reliable and sustainable primary and backup communication systems (e.g. satellite phones, mobile devices, landlines, internet connection, pagers, two-way radios)?

- Yes (1)
- No (2)
- Don't know ***Please consult with your colleagues to avoid using this option, if possible.*** (3)

Display This Question:

If Do you have availability of reliable and sustainable primary and backup communication systems (e.... = Yes

Q5.2 Which options are present at your institution?

- Landlines (1)
- Two-way radios (2)
- Mobile phones (3)
- Satellite phones (4)
- Internet connection (5)
- Pagers (6)
- Web-based communication (7)

Q5.3 Is there access to an updated contact list?

- Yes (1)
- No (2)
- Don't know ***Please consult with your colleagues to avoid using this option, if possible.*** (3)

Q5.4 Is there a procedure for appointing a public information spokesperson to coordinate trauma centre communication with the public, media and health authorities?

- Yes (1)
- No (2)
- Don't know ***Please consult with your colleagues to avoid using this option, if possible.*** (3)

Q5.5 Is there a procedure for briefing hospital staff on their roles and responsibilities with the emergency management plan?

- Yes (1)
- No (2)
- Don't know ***Please consult with your colleagues to avoid using this option, if possible.*** (3)

End of Block: Communication

Start of Block: Triage

Q6.1 Does your institution have a mass casualty triage protocol that follows internationally accepted principles and guidelines?

- Yes (1)
- No (2)
- Don't know ***Please consult with your colleagues to avoid using this option, if possible.*** (3)

Q6.2 Does your institution have a contingency site for receipt and triage of mass casualties?

- Yes (1)
- No (2)
- Don't know ***Please consult with your colleagues to avoid using this option, if possible.*** (3)

Q6.3 Does your institution have mechanisms in place for identifying victims and tracking missing persons?

- Yes (1)
- No (2)
- Don't know ***Please consult with your colleagues to avoid using this option, if possible.*** (3)

End of Block: Triage

Start of Block: Safety and security

Q7.1 Does your institution's disaster preparedness plan include appointment of a hospital security team responsible for hospital safety and security activities?

- Yes (1)
- No (2)
- Don't know ***Please consult with your colleagues to avoid using this option, if possible.*** (26)

Q7.2 Does your institution's disaster preparedness plan include procedures for reliable identification of authorised hospital personnel, patients and visitors?

- Yes (1)
- No (2)
- Don't know ***Please consult with your colleagues to avoid using this option, if possible.*** (3)

Q7.3 Does your institution's disaster preparedness plan include procedures for early control of facility access points, triage sites and other areas of patient flow, traffic and parking?

- Yes (1)
- No (2)
- Don't know ***Please consult with your colleagues to avoid using this option, if possible.*** (3)

Q7.4 Can you limit visitor access (i.e. facility lock-down) as appropriate?

- Yes (1)
- No (2)
- Don't know ***Please consult with your colleagues to avoid using this option, if possible.*** (5)

Q7.5 Does your institution have an established area to deal with radioactive, biological and chemical decontamination and isolation?

- Yes (1)
- No (2)
- Don't know ***Please consult with your colleagues to avoid using this option, if possible.*** (3)

End of Block: Safety and security

Start of Block: Surge capacity

Q8.1 Does your institution have a system to calculate the maximal capacity required for patient admission and care based not only on total number of beds required but also on availability of human and essential resources and the adaptability of space for critical care?

- Yes (1)
- No (2)
- Don't know ***Please consult with your colleagues to avoid using this option, if possible.*** (3)

Display This Question:

If Does your institution have a system to calculate the maximal capacity required for patient admiss... = Yes

Q8.2 Indicate the coverage of this system:

○ Municipal (1)

○ Regional (2)

○ Provincial/state (3)

○ Multi-provincial/multi-state (4)

○ National (5)

○ Don't know ***Please consult with your colleagues to avoid using this option, if possible.*** (6)

Display This Question:

If Does your institution have a system to calculate the maximal capacity required for patient admiss... = Yes

Q8.3 Which of these capacities does this system monitor:

▢ Emergency Department (1)

▢ ICU beds (2)

▢ Operating room availability (3)

▢ Overall bed availability (4)

▢ Staff availability (5)

▢ Air medical services (6)

▢ Patient tracking system (7)

▢ Early warning systems (8)

Q8.4 Does your institution's disaster preparedness plan address the need for increased surgical capacity?

○ Yes (1)

○ No (2)

○ Don't know ***Please consult with your colleagues to avoid using this option, if possible.*** (3)

Display This Question:

If Does your institution's disaster preparedness plan address the need for increased surgical capaci... = Yes

Q8.5 Has the surgical surge capacity been tested?

○ Yes (1)

○ No (2)

○ Don't know ***Please consult with your colleagues to avoid using this option, if possible.*** (3)

Display This Question:

If Does your institution's disaster preparedness plan address the need for increased surgical capaci... = Yes

Q8.6 What is the estimated surgical surge capacity?

○ Patients per hour = (5) ____

○ Don't know ***Please consult with your colleagues to avoid using this option, if possible.*** (7)

Q8.7 Does your institution's disaster preparedness plan address the need for increased Emergency Department capacity?

○ Yes (1)

○ No (2)

○ Don't know ***Please consult with your colleagues to avoid using this option, if possible.*** (3)

Display This Question:

If Does your institution's disaster preparedness plan address the need for increased Emergency Depar... = Yes

Q8.8 Has the ED surge capacity been tested?

- Yes (1)
- No (2)
- Don't know ***Please consult with your colleagues to avoid using this option, if possible.*** (3)

Display This Question:

If Does your institution's disaster preparedness plan address the need for increased Emergency Depar... = Yes

Q8.9 What is the estimated ED surge capacity?

- Patients per hour = (4) ____
- Don't know ***Please consult with your colleagues to avoid using this option, if possible.*** (5)

Q8.10 Does your institution's disaster preparedness plan address the need for increased ICU capacity?

- Yes (1)
- No (2)
- Don't know ***Please consult with your colleagues to avoid using this option, if possible.*** (3)

Display This Question:

If Does your institution's disaster preparedness plan address the need for increased ICU capacity? = Yes

Q8.11 Has the ICU surge capacity been tested?

- Yes (1)
- No (2)
- Don't know ***Please consult with your colleagues to avoid using this option, if possible.*** (3)

Display This Question:

If Does your institution's disaster preparedness plan address the need for increased ICU capacity? = Yes

Q8.12 What is the estimated ICU surge capacity?

- Patients per hour = (4) ____
- Don't know ***Please consult with your colleagues to avoid using this option, if possible.*** (5)

Q8.13 Has your institution estimated its decontamination capacity?

- Yes (1)
- No (2)
- Don't know ***Please consult with your colleagues to avoid using this option, if possible.*** (3)

Display This Question:

If Has your institution estimated its decontamination capacity? = Yes

Q8.14 Has the decontamination capacity been tested?

- Yes (1)
- No (2)
- Don't know ***Please consult with your colleagues to avoid using this option, if possible.*** (3)

Display This Question:

If Has your institution estimated its decontamination capacity? = Yes

Q8.15 What is the estimated decontamination capacity? (patients per hour)

- Patients per hour = (4) ____
- Don't know ***Please consult with your colleagues to avoid using this option, if possible.*** (5)

Q8.16 Does your institution’s plan have designated care areas for patient overflow (e.g. auditorium or lobby)?

- Yes (1)
- No (2)
- Don't know ***Please consult with your colleagues to avoid using this option, if possible.*** (3)

Q8.17 Does your institution have a system to increase hospital capacity by outsourcing the care of non-critical patients to appropriate alternative treatment sites?

- Yes (1)
- No (2)
- Don't know ***Please consult with your colleagues to avoid using this option, if possible.*** (3)

Q8.18 Does your institution have a contingency plan for interfacility patient transfer should traditional methods of transportation become unavailable?

- Yes (1)
- No (2)
- Don't know ***Please consult with your colleagues to avoid using this option, if possible.*** (3)

Q8.19 Can your institution sustain operations at maximum occupancy for 72 hours or more during a mass casualty event?

- Yes (1)
- No (2)
- Don't know ***Please consult with your colleagues to avoid using this option, if possible.*** (3)

Display This Question:

If Can your institution sustain operations at maximum occupancy for 72 hours or more during a mass c... = No

Q8.20 How long can your institution sustain peak operations?

◯ Time in hours = (4) ____

◯ Don't know **Please consult with your colleagues to avoid using this option, if possible.*** (5)

Display This Question:

*If Can your institution sustain operations at maximum occupancy for 72 hours or more during a mass c... != Don't know <i>**Please consult with your colleagues to avoid using this option, if possible.** </i>*

Q8.21 Has this been tested?

◯ Yes (1)

◯ No (2)

◯ Don't know ***Please consult with your colleagues to avoid using this option, if possible.*** (3)

End of Block: Surge capacity

Start of Block: Continuity of essential services

Q9.1 Does your institution have availability of appropriate back-up arrangements for essential life lines including water, power and oxygen?

◯ Yes (1)

◯ No (2)

◯ Don't know ***Please consult with your colleagues to avoid using this option, if possible.*** (3)

Display This Question:

If Does your institution have availability of appropriate back-up arrangements for essential life li... = Yes

Q9.2 For how many days does your institution have back-up supplies for?

____

Q9.3 Does your institution have stored resources in case of a mass casualty event?

- Yes (1)
- No (2)
- Don't know ***Please consult with your colleagues to avoid using this option, if possible.*** (3)

Display This Question:

If Does your institution have stored resources in case of a mass casualty event? = Yes

Q9.4 Which resources have you stored?

- Water (1)
- Food (2)
- Fuel (3)
- Don't know ***Please consult with your colleagues to avoid using this option, if possible.*** (4)

Q9.5 Does your institution have an established mechanism for accepting donation of materials during a mass casualty incident?

- Yes (1)
- No (2)
- Don't know ***Please consult with your colleagues to avoid using this option, if possible.*** (3)

Q9.6 Does your institution have a system in place for determining and storing the optimal amount of pharmaceuticals, laboratory, operating equipment and blood products for a mass casualty event?

- Yes (1)
- No (2)
- Don't know ***Please consult with your colleagues to avoid using this option, if possible.*** (3)

End of Block: Continuity of essential services

Start of Block: Human Resources

Q10.1 Does your institution have a database of staff trained in Emergency management?

- Yes (1)
- No (2)
- Don't know ***Please consult with your colleagues to avoid using this option, if possible.*** (3)

Display This Question:

If Does your institution have a database of staff trained in Emergency management? = Yes

Q10.2 Check all that apply:

- Physicians/surgeons (1)
- Nurses (2)
- Allied health personnel (3)
- Administrative staff (4)
- Hospital management (5)
- Engineers (6)
- Security (7)
- Ancillary staff (8)
- Bed managers (9)

Display This Question:

If Does your institution have a database of staff trained in Emergency management? = Yes

Q10.3 Is the database maintained?

◯ Yes (1)

◯ No (2)

◯ Don't know ***Please consult with your colleagues to avoid using this option, if possible.*** (4)

Q10.4 Does your institution have a training and education plan available for staff involved in mass casualty situations?

◯ Yes (1)

◯ No (2)

◯ Don't know ***Please consult with your colleagues to avoid using this option, if possible.*** (3)

Q10.5 Does your institution have a system for recruiting and training additional staff according to anticipated need?

◯ Yes (1)

◯ No (2)

◯ Don't know ***Please consult with your colleagues to avoid using this option, if possible.*** (3)

Q10.6 Does your institution have a system to ensure the availability of multidisciplinary psychosocial support teams that include social workers, counsellors, interpreters and clergy for the families of staff and patients?

◯ Yes (1)

◯ No (2)

◯ Don't know ***Please consult with your colleagues to avoid using this option, if possible.*** (3)

End of Block: Human Resources

Start of Block: Logistics and supply managament

Q11.1 Has your institution developed and maintained an updated inventory of all equipment, supplies and pharmaceuticals?

- Yes (1)
- No (2)
- Don't know ***Please consult with your colleagues to avoid using this option, if possible.*** (3)

Q11.2 Does your institution have a system to ensure the continuous provision of essential medications and supplies during a mass casualty event?

- Yes (1)
- No (2)
- Don't know ***Please consult with your colleagues to avoid using this option, if possible.*** (3)

Q11.3 Does your institution have contingency agreements with vendors to ensure the procurement and prompt delivery of equipment, supplies and other resources in times of shortage?

- Yes (1)
- No (2)
- Don't know ***Please consult with your colleagues to avoid using this option, if possible.*** (3)

Q11.4 How many operating theatres does your institution have?

- Total number = (4) ____
- Don't know ***Please consult with your colleagues to avoid using this option, if possible.*** (5)

Q11.5 How many plain XR machines does your institution have?

○ Total number = (4) ____

○ Don't know ***Please consult with your colleagues to avoid using this option, if possible.*** (5)

Q11.6 How many CT scanners does your institution have?

○ Total number = (4) ____

○ Don't know ***Please consult with your colleagues to avoid using this option, if possible.*** (5)

Q11.7 How many ICU beds does your institution have?

○ Total number = (4) ____

○ Don't know ***Please consult with your colleagues to avoid using this option, if possible.*** (5)

End of Block: Logistics and supply managament

Start of Block: Post-disaster recovery

Q12.1 Does your institution's disaster preparedness plan include provision of a post-action report to hospital administration, emergency managers and appropriate stakeholders that includes an incident summary, a response assessment and an expenses report?

○ Yes (1)

○ No (2)

○ Don't know ***Please consult with your colleagues to avoid using this option, if possible.*** (3)

Q12.2 Does your institution have a plan for professionally conducted debriefing for staff within 24-72 hours after the occurrence of a mass casualty event?

- Yes (1)
- No (2)
- Don't know ***Please consult with your colleagues to avoid using this option, if possible.*** (3)

Q12.3 Does your institution have a post-disaster employee recovery assistance programme?

- Yes (1)
- No (2)
- Don't know ***Please consult with your colleagues to avoid using this option, if possible.*** (3)

End of Block: Post-disaster recovery

Start of Block: Assistance

Q13.1 Did you consult with colleagues to complete this survey?

- Yes (1)
- No (2)

Display This Question:

If Did you consult with colleagues to complete this survey? = Yes

Q13.2 Who did you consult with?

____

End of Block: Assistance

Start of Block: Submission Warning

Q14.1 Thank you for taking the time to complete this survey.
This is the last question, when the progress button is pressed your survey link will be de-activated and you will not be able to re-access the survey or change any responses.
Please ensure you have completed as much of the survey as possible before clicking the progress button below.
Thank you for your time.

End of Block: Submission Warning
